# A rare case of subclavian steal phenomenon: when a dialysis arm arteriovenous fistula robs the brain

**DOI:** 10.1055/s-0046-1817039

**Published:** 2026-02-27

**Authors:** Leonardo Furtado Freitas, Tate Hodges, Charif Sidani, Kevin J. Abrams

**Affiliations:** 1 Baptist Health South Florida, Radiology Associates of South Florida, Department of Radiology, Miami FL, United States.; 2 Florida International University, Herbert Wertheim College of Medicine, Miami FL, United States.

**Figure 1:**
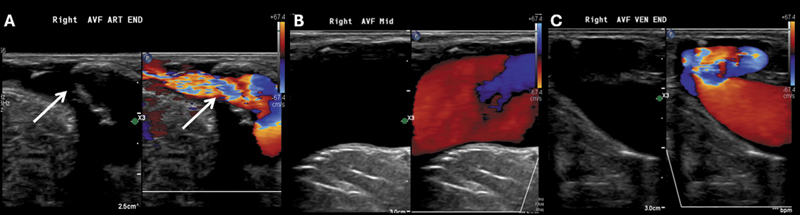
Color Doppler ultrasound images demonstrating the dialysis-related arteriovenous fistula in the right upper extremity, including the arterial (A), anastomotic (B), and venous (C) segments. The fistula was patent, with less than 50% stenosis in the arterial segment, as evidenced by color aliasing (white arrows).

**Figure 2:**
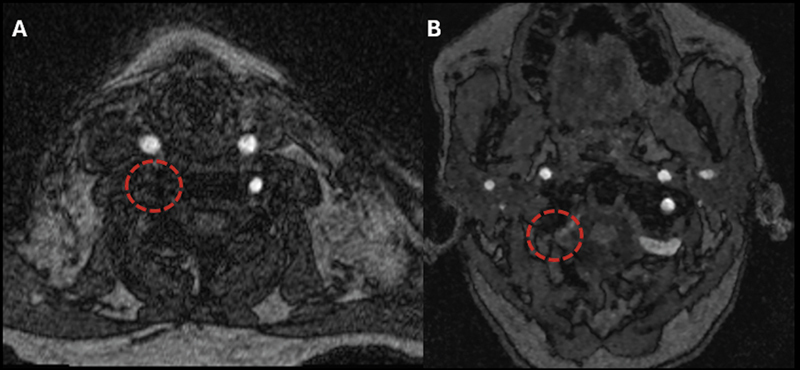
Magnetic resonance angiography of the cervical (A) and intracranial (B) arteries using three-dimensional (3D) time-of-flight (TOF) sequence. The absence of flow-void signal in the cervical and intracranial segments of the right vertebral artery (red dashed circles) suggested slow flow, absence of flow, or retrograde flow from a superior-to-inferior direction.

A 79-year-old woman on hemodialysis via a right arm arteriovenous fistula (AVF), as shown in Figure 1 , presented with recurrent falls and transient dizziness. Neurovascular imaging ( Figures 2 3 ) revealed reversed flow in the right vertebral artery without subclavian artery stenosis, along with aneurysmal dilation of the right subclavian and axillary vessels. This case illustrates a rare subclavian steal phenomenon in the absence of arterial stenosis, highlighting the importance of recognizing dialysis access-related hemodynamic steal in patients presenting with vertebrobasilar symptoms. 1 2 3

**Figure 3:**
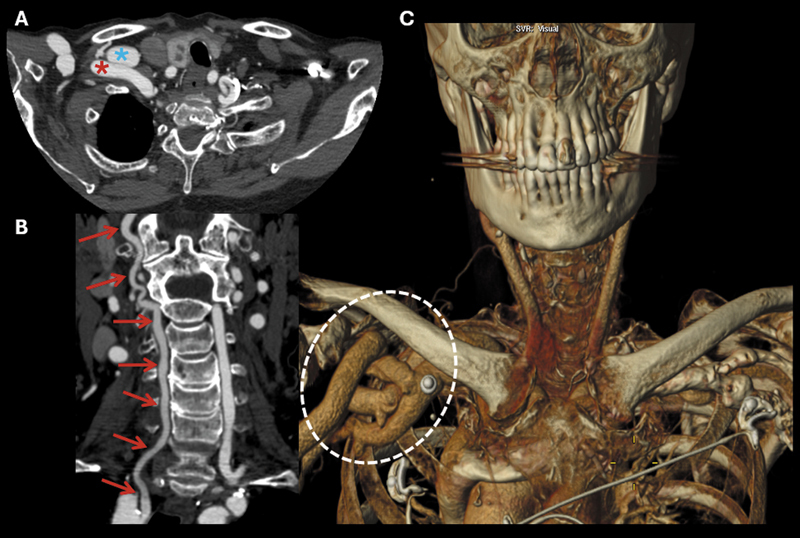
Cervical arterial computed tomography (CT) angiography. Axial (A), coronal (B), and volume-rendering (VR) reconstruction (C). Aneurysmal dilation of the right subclavian artery (red asterisk) and vein (blue asterisk). Patency of the entire right cervical vertebral artery (red arrows), with relatively reduced opacification compared to the contralateral side. On the VR image, the aneurysmal dilations of the right subclavian and axillary vessels are more conspicuous (white dashed circle), related to the presence of the dialysis-related arteriovenous fistula (AVF) in the right upper extremity.
